# The Winning of Wealth

**Published:** 1881-07

**Authors:** J. G. Holland


### THE WINNING OF WEALTH.

WE BELIEVE the winning of wealth to be a perfectly legitimate pursuit. Wealth has great and beneficent uses, and the world would go very slowly if money could not be accumulated in wise and enterprising hands; but wealth may be used to make all men near it prosperous and happy, or it may be used to make them poor and miserable. When a rich man is only excited by his wealth with the desire to be richer, and goes on to exact larger profits and to grind the faces of the poor, in order that he may be superfluously rich, he becomes inhuman and unchristian. The Christian use of wealth is what we need in this country and in all countries. It is not that wealth does not give in charity. It is not that wealth is not sufficiently taxed for the support of those who are wrecked in health or fortune, but it is that weath does not give the people a chance to escape from poverty; that it does not share its chances with the poor, and point the pathway for the poor toward prosperity. As a rule, wealth is only brotherly toward wealth, and the poor man feels himself cut off from sympathy with those who have the power of winning money. We may rest assured of one thing, namely, that the poor in the future will insist on being recognized. If they are not recognized—if they are ignored in the mad greed for wealth at any cost to them—they will make the future a troubled and terrible one for our children and our children's children—*J. G. Holland, in Scribner for July.*

